# 6mA-StackingCV: an improved stacking ensemble model for predicting DNA N6-methyladenine site

**DOI:** 10.1186/s13040-023-00348-8

**Published:** 2023-11-27

**Authors:** Guohua Huang, Xiaohong Huang, Wei Luo

**Affiliations:** 1 School of Information Technology and Administration, Hunan University of Finance and Economics, Changsha, China; 2 College of Information Science and Engineering, Shaoyang University, Shaoyang, Hunan 422000 China

**Keywords:** Cross validation, Meta-learning, 6mA, DNA methylation, Ensemble learning

## Abstract

DNA N6-adenine methylation (N6-methyladenine, 6mA) plays a key regulating role in the cellular processes. Precisely recognizing 6mA sites is of importance to further explore its biological functions. Although there are many developed computational methods for 6mA site prediction over the past decades, there is a large root left to improve. We presented a cross validation-based stacking ensemble model for 6mA site prediction, called 6mA-StackingCV. The 6mA-StackingCV is a type of meta-learning algorithm, which uses output of cross validation as input to the final classifier. The 6mA-StackingCV reached the state of the art performances in the Rosaceae independent test. Extensive tests demonstrated the stability and the flexibility of the 6mA-StackingCV. We implemented the 6mA-StackingCV as a user-friendly web application, which allows one to restrictively choose representations or learning algorithms. This application is freely available at http://www.biolscience.cn/6mA-stackingCV/. The source code and experimental data is available at https://github.com/Xiaohong-source/6mA-stackingCV.

## Introduction

DNA methylation is one of epigenetic modifications, which refers to a chemical process where the methyl groups are attached to the DNA nucleotide residues. So far, it has been reported that DNA methylation occurs only at two nucleotides: adenine and cytosine. The DNA methylation at the cytosine includes N5-methylcytosine (5mC) [1], Hydroxymethylcytosine (5hmC), and N4-methylcytosine (4mC) [2]. The prevalent methylation of DNA at the adenine is N6-Methyladenine (6mA). The 6mA is transferring the methyl group to the sixth position of the purine ring of adenine by the DNA methyltransferase [3, 4]. The 6mA was considered absent in the eukaryote due to limitations of detection techniques, but have been found over the past 10 years in a limited number of species including Chlamydomonas reinhardti [5], Caenorhabiditis elegans [6], mouse [7], and pig [8]. The 6mA have been proven to play a key role in the gene regulation [4], DNA repair [9, 10], DNA replication [11], and epigenetic memory maintenance [4]. The 6mA was closely associated with such diseases as human esophageal squamous cell carcinoma [12], hepatocellular carcinoma [13], and hypertension [14]. The 6mA was even considered as a potential “epigenetic” mark [15].

Accurately identifying the DNA 6mA sites is of great importance for exploring its mechanism and function. Many techniques have been developed to identify 6mA modification, which can be grouped into two categories: wet and dry methodologies. The wet methodology is to use physic or chemistry-based methods to detect 6mA sites, which includes liquid chromatography coupled with tandem mass spectrometry (LC-MS/MS), single-molecule real-time (SMRT) sequencing, 6mA-immunoprecipitation sequencing (6mA-IPseq), restriction enzyme-based sequencing (6mA-REseq) [4, 16]. The 6mA-IPseq is of low sensitivity, low specificity, as well as high false positive rate, and requires high-quality DNA sample without bacterial contamination [16]. The 6mA-REseq is confined to specific restriction site and is of high false positive rate. The LC-MS/MS is of high sensitivity and specificity, but is sensitive to experimental condition and hence is difficult to conduct. The SMRT is of high sensitivity, and is able to detect single-base resolution, but is of relatively low specificity, and easy to confuse 6mA and 1mA. The modifications of flanking cytosine also interfere with the identification of 6mA in the LC-MS/MS [16]. The dry methodology refers to techniques which employ computational methods to recognize or identify the 6mA sites. With the development of artificial intelligence, the dry methodology is increasingly attracting more and more attention. Over the past 10 years, no less than 10 dry methodologies have been developed to identify 6mA sites [3, 17–31].

The representations of DNA N6-methyladenine and the machine learning algorithms are two key factors to determine the predictive accuracy. The machine learning algorithms are classified into the traditional machine learning and the deep learning. The former includes support vector machine (SVM), logistic regression, random forest, multilayer perceptron, and naïve Bayes, which exhibited high performance especially for small samples. The deep learning is actually a deep multiple-layer neural network with specific architectures. Besides the traditional fully-connected network, some network architectures have been proposed, such as long short term memory (LSTM) [32], convolution neural network (CNN) [33], residual network [34], and self-attention [35]. The predictive accuracy of the deep learning heavily depends on the number of training samples. The small samples are easy to make the deep learning model be overfitting. The representations used to predict DNA N6-methyladenine included the One-hot encoding [36, 37], accumulated nucleotide frequency (ANF) [38], enhanced nucleic acid composition (ENAC) [39], composition of K-spaced nucleic acid pairs (CKSNAP) [39], dinucleotide composition (DNC) [40], trinucleotide composition (TNC) [41], nucleotide chemical property (NCP) [38], and pseudo dinucleotide composition (PseDNC) [42]. Some representations performed well, and some performed poorly for the same learning algorithm. For example, One-hot, NCP, EIIP and ENAC showed better performance than the TNC, CKSNAP, PseDNC, DNC and NAC in the 6mA-Finder’s experiments [23]. One-hot showed better performance than EIIP, which was better than dinucleotide One-hot encoding, k-mer composition and k-space spectral nucleotide composition in the i6mA‑Fuse experiments [22]. Single representation was insufficient to characterize DNA N6-methyladenine sequences. A single representation might contain noise to a certain extent, while combining multiple representations might potentially overwhelm some informative representations. Meta-learning is to learn to learn, which learns from output of the classifiers. Meta-learning is a potential solution to these questions such as getting rid of noise, depending on the large number of training samples. Xu et al. used output probabilities of 7 classical machine learning algorithms as representation and construct further the final logistic regression classifier for DNA 6mA prediction [23]. Khanal et al. [25] selected 210 optimal representations from 1570 original representations by the recursive feature elimination with cross-validation, and then constructed a support vector machine-based classifier for 6mA prediction, which took output of four classifiers as input. Hasan et al. [26] used the learning output of 30 classifiers as the input to meta classifiers which was formed by combining 5 categories of representations and 6 classic machine learning algorithms respectively. These meta-learning methods obtained the state of the art performance. We proposed an improved stacking ensemble model for predicting DNA N6-methyladenine site, which is called 6mA-StackingCV. The 6mA-StackingCV used the cross validation to construct multiple classifiers, which improved the robustness and flexibility.

## Results

### Feature selection

Besides one-hot encoding and EIIP, we computed five categories of popular representation of nucleotide sequences, i.e., the NCP, kmer (k = 3), NAC, ENAC, and ANF. The NCP is similar to One-hot encoding. Difference between them lies that the NCP employed chemical properties [38]. According to the ring structure, the nucleotides are grouped into Purine and Pyrimidine. The former has two rings, while the latter has only a ring. Adenine (A) and guanine (G) are classified as purines, while cytosine (C) and thymine (T) are classified as pyrimidines. Nucleotides can also be divided into two functional groups: amino and keto. A and C belong to the amino functional group, while G and T belong to the keto functional group. The nucleotides are divided into strong and weak Hydrogen Bond. G and C fall into strong Hydrogen Bond, while A and T into weak Hydrogen Bond. The NCP integrates the chemical property into one-hot encoding. Each nucleotide ${N}_{i}$ is represented as a 3-dimensional one-hot vector ${(X}_{i}{Y}_{i}{Z}_{i})$, where

$$\begin{array}{lll}\boldsymbol{X_i}=\left\{\begin{array}{ll}\boldsymbol{1},\,\boldsymbol{if\,N_i\epsilon}\left\{\boldsymbol{A,G}\right\}\\\boldsymbol{0},\,if\,\boldsymbol{N_i\epsilon}\,\left\{\boldsymbol{C,T}\right\}\end{array}\right.\\\boldsymbol{Y_i}=\left\{\begin{array}{ll}\boldsymbol{1},\,\boldsymbol{if}\,\boldsymbol{N_{i}\epsilon}\left\{\boldsymbol{A,C}\right\}\\\boldsymbol{0},\,\boldsymbol{if}\,\boldsymbol{N_{i}\epsilon}\left\{\boldsymbol{G,T}\right\}\end{array}\right.{\mathbf{,}\,\mathbf{and}}\\\boldsymbol{Z_i}=\left\{\begin{array}{ll}\boldsymbol{1},\,\boldsymbol{if}\,\boldsymbol{N_{i}\epsilon}\left\{\boldsymbol{A,T}\right\}\\\boldsymbol{0},\,\boldsymbol{if}\,\boldsymbol{N_{i}\epsilon}\left\{\boldsymbol{C,G}\right\}\end{array}\right.\end{array}$$

That’s to say, A is encoded into (1,1,1), C into (0,1,0), G into (1,0,0), and T into (0,0,1). Kmer refers to the occurrence frequencies of k continuous nucleotides. The NAC refers to the occurrence frequency of single nucleotide. ENAC [43] is defined as occurrence frequency of nucleotides in the sliding windows from 5′ to 3’, which is computed by

1 $$\varvec{V}=\left[\frac{{\varvec{N}}_{\varvec{A},\varvec{w}\varvec{i}\varvec{n}1}}{\varvec{S}},\frac{{\varvec{N}}_{\varvec{C},\varvec{w}\varvec{i}\varvec{n}1}}{\varvec{S}},\frac{{\varvec{N}}_{\varvec{G},\varvec{w}\varvec{i}\varvec{n}1}}{\varvec{S}},\frac{{\varvec{N}}_{\varvec{T},\varvec{w}\varvec{i}\varvec{n}1}}{\varvec{S}},\dots ,\frac{{\varvec{N}}_{\varvec{G},{\varvec{w}\varvec{i}\varvec{n}}_{\varvec{L}-\varvec{S}+1}}}{\varvec{S}},\frac{{\varvec{N}}_{\varvec{T},{\varvec{w}\varvec{i}\varvec{n}}_{\varvec{L}-\varvec{S}+1}}}{\varvec{S}}\right]$$

where S is size of the sliding window and S is equal to 5, ${N}_{A,win1}$ denotes occurring number of A in the first window, ${N}_{C,win1}$ the occurring number of C, and so on. The ANF [38, 44] is computed by

2 $${\varvec{d}}_{\varvec{i}}=\frac{1}{\left|{\varvec{S}}_{\varvec{i}}\right|}\sum\nolimits_{\varvec{j}=1}^{\varvec{i}}{\varvec{f}}_{{\varvec{s}}_{\varvec{i}}}({\varvec{s}}_{\varvec{j}})$$

Where ${s}_{i}$ stands for the i-th nucleotide residue, ${S}_{j}$ refers to the first j nucleotide residues in the sequence. ${f}_{{s}_{i}}\left({s}_{j}\right)$ is computed by

3 $${\varvec{f}}_{{\varvec{s}}_{\varvec{i}}}\left({\varvec{s}}_{\varvec{j}}\right)=\left\{\begin{array}{cc}1& {\varvec{s}}_{\varvec{j}}={\varvec{s}}_{\varvec{i}}\\ 0& \varvec{o}\varvec{t}\varvec{h}\varvec{e}\varvec{r}\end{array}\right.$$

The ANF reflects distribution of positions and nucleotides. A sequence with N nucleotide residues has N ANF features.

We used XGBoost as the learning algorithm and employed hold out to examine performance of feature’ distinguishing between 6mA and non-6mA. The hold out is to split the training set into two parts, one for training and another for validation. We set the ratio of splitting the training set as 8 to 2. We repeated hold out test five times. The average performance and 95% confidence intervals were listed in Table 1. The One-hot encoding performed best, followed by the NCP, then by EIIP, and then by the ENAC, whose average ACC and the 95% confidence intervals were more than 0.93. Three categories of features, namely Kmer, NAC, and ANF, performed worse with less than 0.81 average accuracies. We removed these three categories of features. Next, we further tested the combination of One-hot encoding with other single. The combination of One-hot encoding with EIIP performed best, reaching an average ACC of 0.9469. Then, we continued to add the NCP and the ENAC respectively for testing. As shown in Table 2, three categories of features performed worse than combination of One-hot encoding with EIIP. Therefore, we stopped adding the features. The optimal representations of DNA 6mA sequences were One-hot encoding and EIIP.

**Table 1:** Predictive performance of single category of feature

Feature category	ACC (95%CI)	AUC (95%CI)
NAC	0.5760 (0.5687–0.5834)	0.6134 (0.6043–0.6225)
Kmer(K = 3)	0.6776 (0.6730–0.6822)	0.7447 (0.7386–0.7509)
ANF	0.8005 (0.7959–0.8051)	0.8795 (0.8768–0.8823)
ENAC	0.9348 (0.9316–0.9379)	0.9799 (0.9781–0.9818)
One-hot	0.9461 (0.9428–0.9495)	0.9847 (0.9834–0.9860)
NCP	0.9447 (0.9420–0.9474)	0.9840 (0.9831–0.9849)
EIIP	0.9427 (0.9395–0.9458)	0.9833 (0.9822–0.9844)

**Table 2:** Predictive performance of feature combinations

Feature category	ACC (95%CI)	AUC (95%CI)
One-hot + NCP	0.9468 (0.9433–0.9502)	0.9849 (0.9834–0.9863)
One-hot + EIIP	0.9469 (0.9430–0.9508)	0.9850 (0.9833–0.9867)
One-hot + ENAC	0.9459 (0.9431–0.9487)	0.9848 (0.9832–0.9863)
One-hot + EIIP + NCP	0.9458 (0.9424–0.9492)	0.9845 (0.9834–0.9857)
One-hot + EIIP + ENAC	0.9462 (0.9431–0.9493)	0.9849 (0.9833–0.9865)

### Model selection

We used hold-out to optimize the model. We employed the backward searching strategy. Firstly, we used six popular classifiers (Xgboost, LightGBM, Gradient boosting, random forest, logistic regression, and decision tree) in the first layer, and fixed support vector machine in the second layer. We conducted hold-out test. Then, we removed a classifier each time. The increased ACC meant that the removed classifier contributed negatively to the performance, and was not used in the subsequent experiments. On the contrary, the decreased ACC meant that the removed classifier contributed positively to the performance, and was preserved in the subsequent experiments. We performed above two steps repeatedly until there was not new combination. The performances of all combinations of classifiers were listed in Table 3. Obviously, The combination of the XGBoost, the Gradient boosting, and the LightGBM obtained the best ACC.

**Table 3:** Performance of combining different classifiers in the first layer

First- layer classifiers	ACC (95%CI)	AUC (95%CI)
XGBoost, Gradient boosting, LightGBM, random forest, logistic regression, decision tree	0.9484 (0.9440–0.9528)	0.9588 (0.9551–0.9624)
XGBoost, Gradient boosting, LightGBM, random forest, logistic regression	0.9486 (0.9444–0.9527)	0.9589 (0.9557–0.9622)
XGBoost, Gradient boosting, LightGBM, random forest	0.9487 (0.9446–0.9529)	0.9605 (0.9573–0.9637)
XGBoost, Gradient boosting, LightGBM	0.9488 (0.9445–0.9531)	0.9616 (0.9586–0.9646)
XGBoost, Gradient boosting	0.9469 (0.9430–0.9509)	0.9582 (0.9545–0.9620)
XGBoost, LightGBM	0. 9477 (0.9438–0.9517)	0.9605 (0.9583–0.9627)
Gradient boosting, LightGBM	0.9449 (0.9409–0.9490)	0.9597 (0.9572–0.9621)

After fixing XGBoost, Gradient boosting and Lightgbm in the first layers, we optimized the second layer. We placed random forest, logistic regression, decision tree, XGBoost, Gradient boosting, LightGBM, and SVM in the second layer respectively, and then conducted hold-out test. The performance was listed in Table 4. The LightGBM and the SVM reached the average ACC of 0.9490 and 0.9488, respectively, exceeding all other methods. The predictive accuracies of SVM and the LightGBM were be close to each other. Therefore, we used the XGBoost, Gradient boosting, and LightGBM in the first layer and the SVM in the second layer to construct 6mA-StackingCV.

**Table 4:** Performance of different classifiers in the second layer

Second-layer classifier	ACC (95%CI)	AUC (95%CI)
random forest	0.9448 (0.9425–0.9471)	0.9817 (0.9794–0.9839)
logistic regression	0.9483 (0.9446–0.9521)	0.9855 (0.9840–0.9869)
decision tree	0.9172 (0.9132–0.9212)	0.9172 (0.9132–0.9212)
XGBoost	0.9476 (0.9447–0.9505)	0.9849 (0.9836–0.9862)
Gradient boosting	0.9486 (0.9455–0.9517)	0.9853 (0.9836–0.9870)
LightGBM	0.9490 (0.9455–0.9526)	0.9854 (0.9839–0.9870)
SVM	0.9488 (0.9445–0.9531)	0.9616 (0.9586–0.9646)

### Comparison with existing methods

With development of artificial intelligence, more and more attentions have been paid to computational methods for 6mA identification. Over the past decades, more than ten computational methods have been developed to predict 6mA sites. We compared the 6mA-StackingCV with these existing methods by three independent tests. One independent test was to test 6mA-StackingCV for ability to predict Rosaceae 6mA sites, and other two independent tests are to test 6mA-StackingCV for ability to predict 6mA sites across species. As shown in Table 5, the 6mA-StackingCV obtained the state of the art performances, outperforming all the methods in the Rosaceae independent test. For example, the 6mA-StackingCV increased the ACC by 0.005, the MCC by 0.011, SN by 0.004, and SP by 0.007 over the i6mA-vote [45] which is the latest method for 6mA prediction published in 2022. Although the MM-6mAPred [18] was slightly better than the 6mA-StackingCV in SN, the latter was much better than the former in other respects including ACC and MCC. In the Arabidopsis independent test, the 6mA-StackingCV was competitive with these methods. Except the Meta-i6mA [26] and the i6mA-vote [45], the 6mA-StackingCV was still the best in terms of ACC. In the Rice independent test, the 6mA-StackingCV was inferior to Meta-i6mA [26], i6mA-Fuse_FV [22], i6mA-stack_FV [25], and i6mA-vote [45], but was superior to i6mA-Fuse_RC [22], i6mA-Pred [28], iDNA6mA-Rice [27], MM-6mAPred [18], and 6mA-Finder [23]. We retrieved two datasets of 6mA from the website: https://github.com/YuXuan-Glasgow/SNN6mA [46]. One was from Arabidopsis thaliana (A. thaliana), which contained 19,632 6mA sites and 19,632 non-6mA sites, and another was from Drosophila melanogaster (D. melanogaster), which comprised 10,653 6mA sites and 10,653 non-6mA sites. Each dataset was divided into the training and the testing datasets at the ratio of 9 to 1. The training datasets were used to train the 6mA-StackingCV, and the testing datasets were used to validate effectiveness and efficiency of the 6mA-StackingCV. As shown in Table 6, the 6mA-StackingCV was inferior to the SNN6mA, but obtained more than 0.91 ACCs. Table 6 also showed performance of other 4 methods. Obviously, except the SNN6mA, the 6mA-StackingCV performed best in terms of ACC. The 6mA-StackingCV elevated the ACC by 0.002 over LA6mA [47], by 0.027 over the AL6mA [47], by 0.045 over the iDNA6mA [48] by 0.033 over the i6mA-DNC [49] for the A. thaliana testing dataset. The 6mA-StackingCV raised the ACCs by 0.039 over the AL6mA, by 0.005 over the LA6mA, by 0.054 over the iDNA6mA, and by 0.024 over the i6mA-DNC for the D. melanogaster testing dataset.

**Table 5:** Comparison with state of the art methods

Species	Methods	ACC	MCC	SN	SP
Rosaceae	Meta-i6mA*	0.953	0.905	0.954	0.951
	i6mA-Fuse_FV*	0.943	0.887	0.924	0.962
	i6mA-Fuse_RC*	0.893	0.786	0.890	0.895
	i6mA-stack_FV*	0.928	0.856	0.928	0.927
	i6mA-stack_RC*	0.899	0.798	0.920	0.877
	i6mA-Pred*	0.840	0.684	0.897	0.782
	iDNA6mA-Rice*	0.878	0.764	0.951	0.805
	MM-6mAPred*	0.873	0.758	0.961	0.785
	6mA-Finder*	0.846	0.701	0.928	0.764
	i6mA-vote*	0.955	0.909	0.955	0.954
	6mA-StackingCV	0.960	0.920	0.959	0.961
Rice	Meta-i6mA*	0.880	0.768	0.957	0.802
	i6mA-Fuse_FV*	0.890	0.781	0.921	0.859
	i6mA-Fuse_RC*	0.775	0.571	0.907	0.644
	i6mA-stack_FV*	0.876	0.756	0.938	0.815
	i6mA-stack_RC*	0.813	0.640	0.915	0.712
	i6mA-Pred*	0.791	0.592	0.878	0.705
	iDNA6mA-Rice*	0.755	0.561	0.960	0.547
	MM-6mAPred*	0.834	0.689	0.958	0.710
	6mA-Finder*	0.809	0.636	0.928	0.690
	i6mA-vote*	0.882	0.774	0.961	0.803
	6mA-StackingCV	0.845	0.710	0.963	0.726
Arabidopsis	Meta-i6mA*	0.787	0.600	0.636	0.936
	i6mA-Fuse_FV*	0.749	0.542	0.545	0.949
	i6mA-Fuse_RC*	0.757	0.534	0.615	0.897
	i6mA-stack_FV*	0.770	0.570	0.604	0.933
	i6mA-stack_RC*	0.751	0.514	0.634	0.865
	i6mA-Pred*	0.730	0.462	0.679	0.780
	iDNA6mA-Rice*	0.734	0.473	0.655	0.812
	MM-6mAPred*	0.765	0.531	0.784	0.747
	6mA-Finder*	0.724	0.448	0.741	0.706
	i6mA-vote*	0.798	0.617	0.666	0.929
	6mA-StackingCV	0.782	0.576	0.677	0.886

The asterisk (*) indicated that the results were from the literature [45]

**Table 6:** Comparison with other 5 existing methods

Methods	Species	SN	SP	ACC	MCC	AUC
AL6mA*	A. thaliana	0.862	0.905	0.884	0.768	0.945
LA6mA*		0.899	0.917	0.909	0.817	0.962
iDNA6mA*		0.843	0.889	0.866	0.733	0.932
i6mA-DNC*		0.846	0.909	0.878	0.757	0.944
SNN6mA*		0.899	0.936	0.916	0.832	0.966
6mA-StackingCV		0.887	0.935	0.911	0.823	0.933
AL6mA*	D. melanogaster	0.840	0.916	0.878	0.758	0.941
LA6mA*		0.909	0.915	0.912	0.824	0.966
iDNA6mA*		0.883	0.843	0.863	0.727	0.937
i6mA-DNC*		0.869	0.917	0.893	0.787	0.947
SNN6mA*		0.911	0.949	0.925	0.851	0.968
6mA-StackingCV		0.899	0.934	0.917	0.834	0.929

The asterisk (*) indicated that the results were from the literature [46]

### Test across species

We further tested ability for the 6mA-StackingCV to predict 6mA site across species. As shown in Table 7, the predicting ability across species varied with the training and the testing species. The trained 6mA-StackingCV by the Rice dataset performed best over the Rosaceae testing dataset. Next, the trained 6mA-StackingCV by the Arabidopsis dataset obtained the second best performance over the Rosaceae testing dataset. The worse cases included the 6mA-StackingCV trained by the Rice dataset and tested by the Arabidopsis dataset, and the 6mA-StackingCV trained by the Rosaceae dataset and tested by the Arabidopsis dataset, with less than 0.8 ACC. The ability across species was asymmetric. The trained 6mA-StackingCV by the Rice dataset obtained an ACC of 0.943 over the Rosaceae dataset. On the contrary, the trained 6mA-StackingCV by the Rosaceae dataset obtained an ACC of 0.845 over the Rice dataset, which was reduced by about 0.1. Thus similar asymmetric phenomena were observed everywhere in Table 7.

**Table 7:** Performance across species

Training species	Testing species	SN	SP	ACC	MCC	AUC
Arabidopsis	Rosaceae	0.938	0.862	0.900	0.803	0.924
	Rice	0.949	0.655	0.802	0.632	0.831
Rice	Rosaceae	0.926	0.960	0.943	0.887	0.955
	Arabidopsis	0.576	0.954	0.766	0.573	0.753
Rosaceae	Rice	0.963	0.726	0.845	0.710	0.845
	Arabidopsis	0.677	0.886	0.782	0.576	0.782

### Features’ contribution analysis

**Fig. 1:**
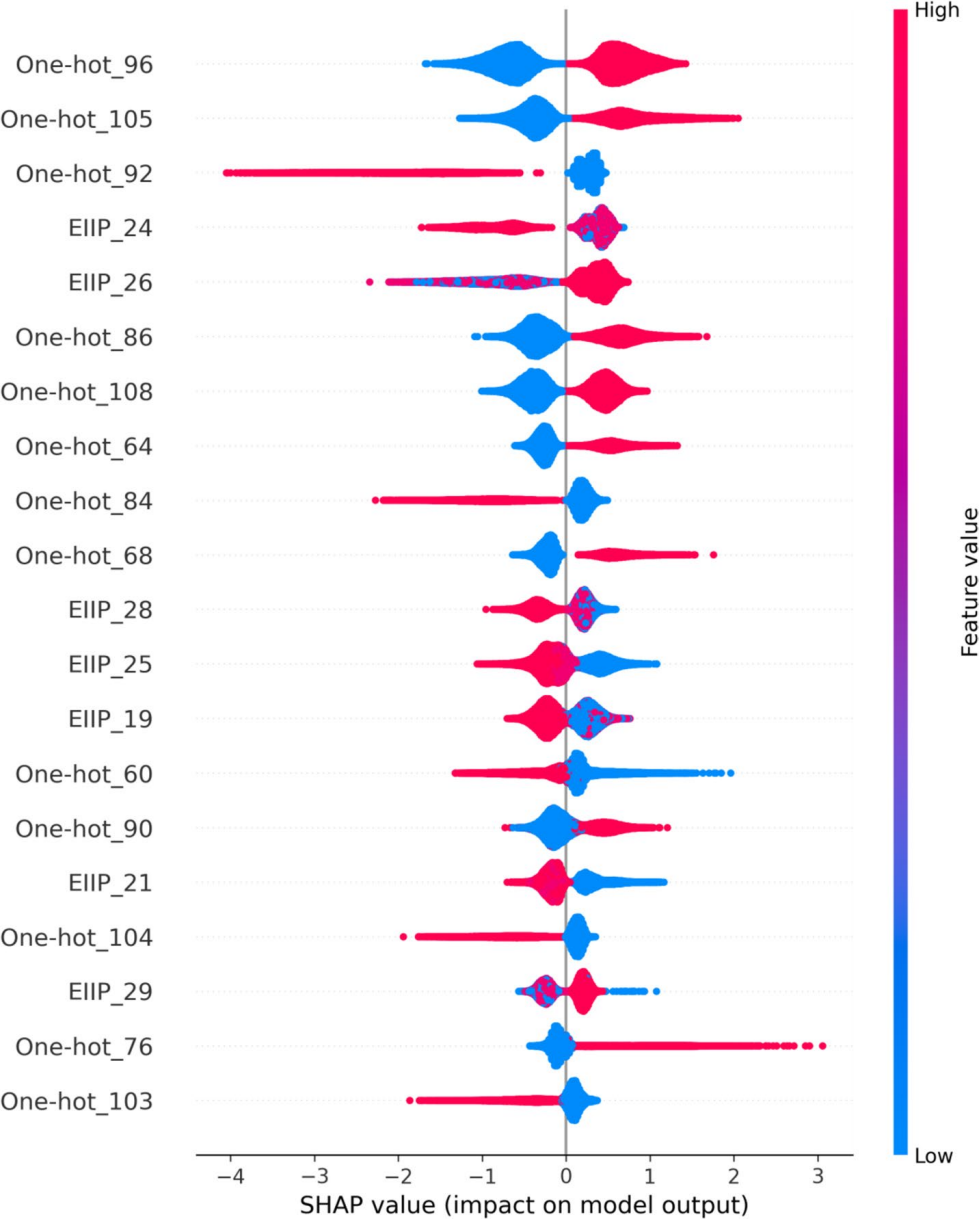
The SHAP value of features. Each point stands for a sample. The red represents larger value, while the blue smaller value of the feature. The larger the SHAP at the x-axis was, the more important the feature contributed to 6mA recognition

We used the SHAP (SHapley Additive exPlanations) [50] to explore feature contribution to 6mA recognition. The SHAP is a game theoretical method with the ability to interpret the output of machine learning model. As shown in Fig. 1, the most important 20 features which influenced the output were ranked from the top to the bottom in the descending order of SHAP value. The higher position the feature was located at, the larger contribution to 6mA recognition it was. For example, the best important feature was one-hot_96, followed by the one-hot_105. The larger the one-hot_96 and the one-hot_105 were, the more probably the output was predicted as 6mA. Conversely, the larger one-hot_92 was feasible to result in non-6mA prediction. Of 20 most important features, 7 EIIP features accounted for more than 1/3, implying its contribution to 6mA recognition. EIIP features included EIIP_19, EIIP_21, EIIP_24, EIIP_25, EIIP_26, EIIP_28, and EIIP_29, indicated that the energy of delocalized electrons of amino acids close to the adenine was a key mark to identify 6mA.

## Discussion

We proposed a cross validation-based stacking ensemble model 6mA-StackingCV for 6mA site prediction. The 6mA-StackingCV reached state of the art performance in the Rosaceae and was superior to i6mA-Fuse_RC [22], i6mA-Pred [28], iDNA6mA-Rice [19], MM-6mAPred [18], and 6mA-Finder [23] across the species. Similar to the 6mA-Finder [23] and meta-i6mA [26], the 6mA-StackingCV was a meta-learning model, which used the output probabilities of several classifiers as input to the final decision. The main difference lay that the 6mA-StackingCV used cross validation to construct different classifiers. If we used all the training data to construct a classifier, the 6mA-StackingCV was identical to the 6mA-Finder [23], the Meta-i6mA [26], and the i6mA-stack [25] in the computing framework despite using different machine learning algorithms and representations. The 6mA-StackingCV outperformed the 6mA-Finder by 0.114 ACC, by 0.219 MCC, by 0.197 SP, and by 0.031 SN in Rosaceae, was superior to the 6mA-Finder by 0.036 ACC, and by 0.074 MCC in the Rice, and exceeded the 6mA-Finder by 0.058 ACC as well as by 0.128 MCC in the Arabidopsis. The difference between the 6mA-StackingCV and the 6mA-Finder [23] was that the former optimized the combinations of classifiers and features. Compared with these methods, the 6mA-StackingCV was robust. As shown in Table 2, some other feature combination also reached approximate performances to combination of One-hot encoding with EIIP. For instance, the combination of One-hot encoding with NCP, the combination of One-hot encoding with ENAC, the combination of One-hot encoding with EIIP and NCP, and the combination of One-hot encoding with EIIP and ENAC reached ACCs of 0.9468, 0.9459, 0.9458, and 0.9462 respectively. The reduction of ACC was no more than 0.001. As shown in Table 3, addition or removal of some classifiers generated little effect on performance. For example, using XGBoost, Gradient boosting, LightGBM, random forest, logistic regression, and decision tree, using gradient boosting and LightGBM, or using XGBoost, Gradient boosting, LightGBM, random forest, and logistic regression in the first layer was of approximately equal performance. Using SVM, random forest, Gradient boosting, XGBoost, LightGBM, or logistic in the second layer was of approximately equal performance (Table 4). The 6mA-StackingCV was also flexible. We respectively selected One-hot + EIIP, One-hot + NCP, and One-hot + EIIP + NCP as representations, and SVM, LightGBM, logistic regression as learning algorithm in the second layer. The performances on the Rosaceae dataset were listed in Table 8. Obviously, the differences of performance were small. This allows one to flexibly construct a 6mA-StackingCV by choosing the appropriate representations and learning algorithms.

**Table 8:** The performance of different classifiers with different representations

Second-layer classifier	Feature	SN	SP	ACC	MCC	AUC
SVM	One-hot + EIIP	0.9589	0.9614	0.9601	0.9203	0.9734
	One-hot + NCP	0.9551	0.9611	0.9581	0.9162	0.9707
	One-hot + ENAC	0.9585	0.9597	0.9591	0.9182	0.9696
	One-hot + EIIP + NCP	0.9542	0.9621	0.9581	0.9163	0.9706
LightGBM	One-hot + EIIP	0.9605	0.9592	0.9599	0.9197	0.9912
	One-hot + NCP	0.9564	0.9603	0.9584	0.9167	0.9907
	One-hot + ENAC	0.9593	0.9575	0.9584	0.9168	0.9909
	One-hot + EIIP + NCP	0.9537	0.9622	0.9579	0.9159	0.9911
logistic regression	One-hot + EIIP	0.9589	0.9611	0.9600	0.9200	0.9913
	One-hot + NCP	0.9563	0.9610	0.9586	0.9173	0.9909
	One-hot + ENAC	0.9600	0.9590	0.9595	0.9190	0.9913
	One-hot + EIIP + NCP	0.9553	0.9607	0.9580	0.9160	0.9909

## Conclusion

We presented an improved stacking ensemble model for predicting DNA N6-methyladenine site. The 6mA-StackingCV was superior to the state of the art methods for Rosaceae, and competitive with those for Arabidopsis and Rice. The 6mA-StackingCV was robust and flexible, benefiting from using cross validation to construct the classifiers. We implemented the 6mA-StackingCV into a user-friendly webserver which is freely available at http://www.biolscience.cn/6mA-stackingCV/. The 6mA-StackingCV was easy to use.

## Materials and methods

### Experimental datasets

High quality dataset is very essential to construct a classifier for precisely identifying DNA 6mA sites. We used the same datasets as the i6mA-vote [45] which were from the Meta-i6mA [26]. Different from Meta-i6mA [26], the i6mA-vote [45] removed the sequences longer than 41 bp and the copy sequences. These datasets are from three species: Rosaceae, Rice, and Arabidopsis. The Rice dataset was compiled by Lv et al. [19], and the Rosaceae and the Arabidopsis datasets were compiled by Hasan et al. [26]. The Rosaceae dataset was further divided into the training and the testing sets at the ratio of 8 to 2. The Rosaceae testing set was used to examine ability to precisely predict 6mA in Rosaceae, while the Rice and the Arabidopsis datasets were used to examine ability to precisely predict 6mA across species. The Rosaceae training set consisted of 29,237 positive and 29,433 negative sequences, the Rosaceae testing set of 7298 positive and 7300 negative sequences, Rice dataset 153,635 positive and 153,629 negative sequences, and the Arabidopsis dataset 31,414 positive and 31,843 negative sequences. The positive sequences referred to the ones containing 6mA sites, while the negative sequences to ones without 6mA sites. All the positive or negative sequences are 41 nucleotide residues.

### One-hot encoding

One-hot encoding is a simple but effective method to encode RNA/DNA/protein sequences. Each character in a sequence is encoded into a vector where only an element is 1 and other are zero. Here, A, C, G, and T are respectively are encoded into (1,0,0,0), (0,1,0,0), (0,0,1,0), and (0,0,0,1).

### EIIP

The EIIP [51, 52] was defined as encoding a character into a digit, i.e., for DNA sequences, A into 0.1260, T into 0.1335, C into 0.1340, and G into 0.0806. The DNA sequence ATTCAGA was encoded by EIIP into (0.1260, 0.1335, 0.1335, 0.1340, 0.1260, 0.0806, 0.1260).

### Stacking ensemble learning with cross validation

**Fig. 2:**
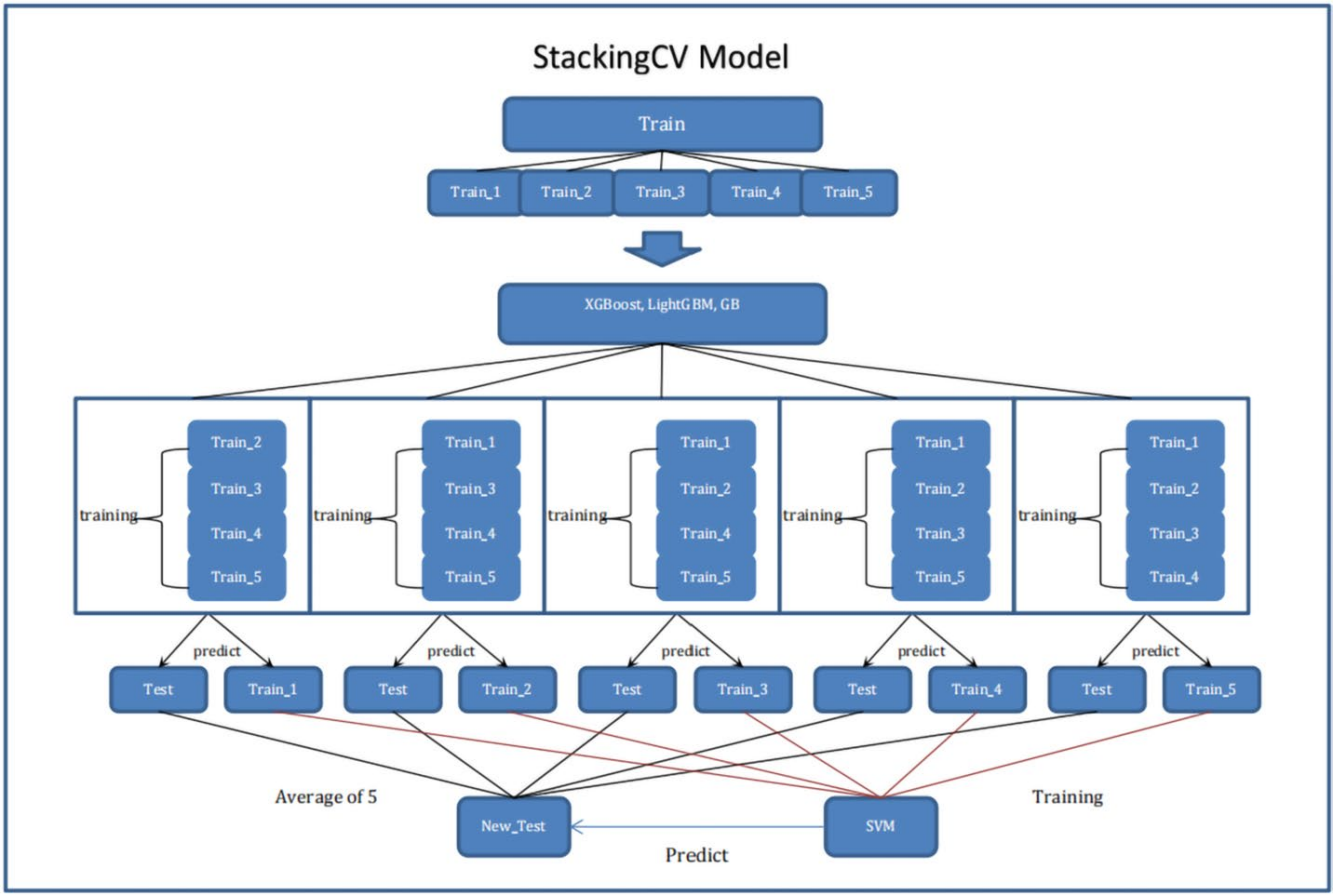
The overview of the proposed 6mA-StackingCV

We used a stacking ensemble model with cross validation for predicting 6mA sites whose idea originated from StackTADB [53], an effective and efficient method for predicting the boundaries of topologically associating domains accurately in fruit flies. The model consisted of two layers. The first layer contained N different base classifiers, and the second layer contained only a classifier. The training set was divided into 5 parts in equal or approximate size. The training process of the stacking ensemble model was described as follows. For each base classifier, we perform 5-fold cross validation over the training set. Therefore, each sample in the training set corresponded to a predicted value. If There were N different base classifiers, so each sample have N predicted value which was further used to train the classifier in the second layer along with its label. An unlabeled encoded sample was predicted by base classifiers trained by 5-fold cross validation, which result in five predicted value. The average over the five predicted value was used as one feature of the sample. N base classifiers yielded N average features, which were further inputted into the final classifier for final decision. Figure 2 showed the schematic diagram of the stack ensemble learning with cross validation.

### Evaluation metrics

To quantitatively measure performance of methods, the following metrics: sensitivity (SN), Specificity (SP), accuracy (ACC), and Matthews’s correlation coefficient (MCC), were used, which were computed by

4 $$\varvec{S}\varvec{N}=\frac{\varvec{T}\varvec{P}}{\varvec{T}\varvec{P} + \varvec{F}\varvec{N}}$$

5 $$\varvec{S}\varvec{P}=\frac{\varvec{T}\varvec{N}}{\varvec{F}\varvec{P} + \varvec{T}\varvec{N}}$$

6 $$\varvec{A}\varvec{C}\varvec{C}=\frac{\varvec{T}\varvec{P} + \varvec{T}\varvec{N}}{\varvec{T}\varvec{P} + \varvec{F}\varvec{N} + \varvec{F}\varvec{P}+ \varvec{T}\varvec{N}}$$

7 $$\varvec{M}\varvec{C}\varvec{C}=\frac{\varvec{T}\varvec{P} \times \varvec{T}\varvec{N} - \varvec{F}\varvec{P} \times \varvec{F}\varvec{N}}{\sqrt{(\varvec{T}\varvec{P} + \varvec{F}\varvec{N})(\varvec{T}\varvec{P} + \varvec{F}\varvec{P})(\varvec{T}\varvec{N} + \varvec{F}\varvec{N})(\varvec{T}\varvec{N} + \varvec{F}\varvec{P})}}$$

Where TP denoted the number of the correctly predicted 6mA samples, TN the number of the correctly predicted non-6mA samples, FP the number of wrongly predicted 6mA sample, and FN the number of wrongly predicted non-6mA samples.

AUC is defined as the area under the Receiver Operating Characteristic (ROC) curve which is drawn by linking true positive rates against false positive rates under various thresholds. The AUC ranges from 0 to 1, with larger values indicating better performance. An AUC of 1 represents perfect prediction, an AUC of 0.5 represents random guess, and an AUC of 0 represents completely reversed prediction.

### 6mA-StackingCV webserver

**Fig. 3:**
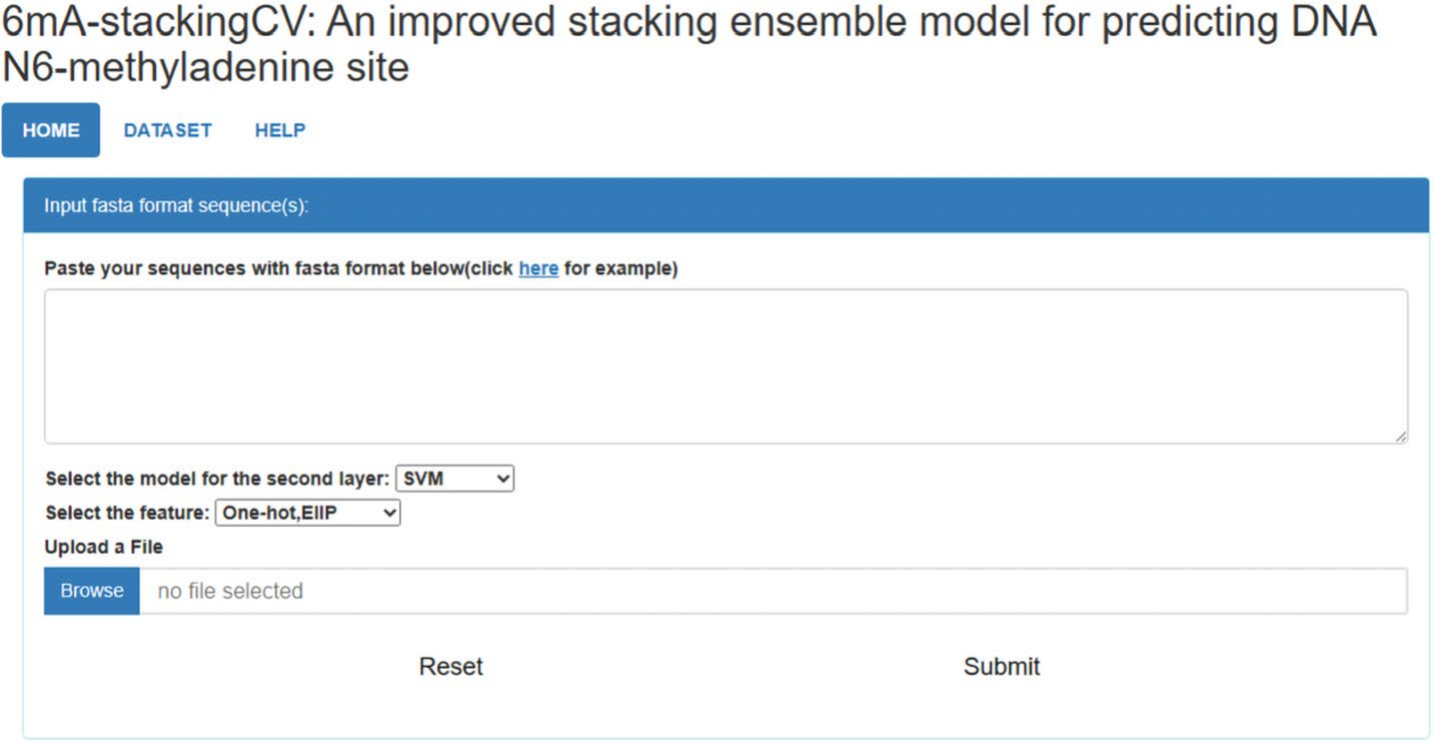
The webserver page of the 6mA-StackingCV

We developed an online webserver to conveniently use the 6mA-StackingCV which is available at http://www.biolscience.cn/6mA-stackingCV/. As shown in Fig. 3, the tool is easy to use. It requires only three steps to complete a prediction. The first step is to upload the DNA sequence in the FASTA format. One can either directly paste the sequence into the text box or upload the file. The web server also provides examples of input sequences. The second step is to select the representations and the learning algorithms. One can click the drop-down menu to select the corresponding representations and learning algorithms. The third step is to click the submit button to conduct a prediction. If one wants to re-upload data, they can click the reset button. The predicted results are returned on an HTML page. The time costed for the prediction is related to the internet speed and the number of uploaded sequences. The web server provided all the experimental datasets for downloading.

## Data Availability

All the experimental data was available at http://www.biolscience.cn/6mA-stackingCV/. The source code was available at https://github.com/Xiaohong-source/6mA-stackingCV.

## References

[1] Breiling A, Lyko F (2015). Epigenetic regulatory functions of DNA modifications: 5-methylcytosine and beyond. Epigenetics Chromatin.

[2] Rodriguez F, Yushenova IA, DiCorpo D, Arkhipova IR (2022). Bacterial N4-methylcytosine as an epigenetic mark in eukaryotic DNA. Nat Commun.

[3] Tang X, Zheng P, Li X, Wu H, Wei D-Q, Liu Y, Huang G (2022). Deep6mAPred: a CNN and Bi-LSTM-based deep learning method for predicting DNA N6-methyladenosine sites across plant species. Methods.

[4] Xie S-Q, Xing J-F, Zhang X-M, Liu Z-Y, Luan M-W, Zhu J, Ling P, Xiao C-L, Song X-Q, Zheng J (2020). N 6-Methyladenine DNA modification in the Woodland Strawberry (Fragaria vesca) Genome reveals a positive relationship with gene transcription. Front Genet.

[5] Fu Y, Luo G-Z, Chen K, Deng X, Yu M, Han D, Hao Z, Liu J, Lu X, Dore LC (2015). N6-methyldeoxyadenosine marks active transcription start sites in Chlamydomonas. Cell.

[6] Greer EL, Blanco MA, Gu L, Sendinc E, Liu J, Aristizabal-Corrales D, Hsu C-H, Aravind L, He C, Shi Y (2015). DNA methylation on N6-adenine in C. Elegans. Cell.

[7] Wu TP, Wang T, Seetin MG, Lai Y, Zhu S, Lin K, Liu Y, Byrum SD, Mackintosh SG, Zhong M (2016). DNA methylation on N 6-adenine in mammalian embryonic stem cells. Nature.

[8] Liu J, Zhu Y, Luo G-Z, Wang X, Yue Y, Wang X, Zong X, Chen K, Yin H, Fu Y (2016). Abundant DNA 6mA methylation during early embryogenesis of zebrafish and pig. Nat Commun.

[9] Pukkila PJ, Peterson J, Herman G, Modrich P, Meselson M (1983). Effects of high levels of DNA adenine methylation on methyl-directed mismatch repair in Escherichia coli. Genetics.

[10] Au KG, Welsh K, Modrich P (1992). Initiation of methyl-directed mismatch repair. J Biol Chem.

[11] Campbell JL, Kleckner N (1990). E. Coli oriC and the dnaA gene promoter are sequestered from dam methyltransferase following the passage of the chromosomal replication fork. Cell.

[12] Chen L, Zhang M, Guo M (2020). DNA N6-methyladenine increased in human esophageal squamous cell carcinoma. Discov Med.

[13] Lin Q, Chen J-w, Yin H, Li M-a, Zhou C-r, Hao T-f, Pan T, Wu C, Li Z-r, Zhu D (2022). DNA N6-methyladenine involvement and regulation of hepatocellular carcinoma development. Genomics.

[14] Guo Y, Pei Y, Li K, Cui W, Zhang D (2020). DNA N6-methyladenine modification in Hypertension. Aging.

[15] Heyn H, Esteller M (2015). An adenine code for DNA: a second life for N6-methyladenine. Cell.

[16] Li H, Zhang N, Wang Y, Xia S, Zhu Y, Xing C, Tian X, Du Y (2022). DNA N6-Methyladenine modification in eukaryotic genome. Front Genet.

[17] Li Z, Jiang H, Kong L, Chen Y, Lang K, Fan X, Zhang L, Pian C (2021). Deep6mA: a deep learning framework for exploring similar patterns in DNA N6-methyladenine sites across different species. PLoS Comput Biol.

[18] Pian C, Zhang G, Li F, Fan X (2020). MM-6mAPred: identifying DNA N6-methyladenine sites based on Markov model. Bioinformatics.

[19] Lv H, Dao F-Y, Guan Z-X, Zhang D, Tan J-X, Zhang Y, Chen W, Lin H (2019). iDNA6mA-Rice: a computational tool for detecting N6-methyladenine sites in rice. Front Genet.

[20] Chen W, Lv H, Nie F, Lin H (2019). i6mA-Pred: identifying DNA N6-methyladenine sites in the rice genome. Bioinformatics.

[21] Huang Q, Zhang J, Wei L, Guo F, Zou Q (2020). 6mA-RicePred: a method for identifying DNA N 6-methyladenine sites in the rice genome based on feature fusion. Front Plant Sci.

[22] Hasan MM, Manavalan B, Shoombuatong W, Khatun MS, Kurata H (2020). i6mA-Fuse: improved and robust prediction of DNA 6 mA sites in the Rosaceae genome by fusing multiple feature representation. Plant Mol Biol.

[23] Xu H, Hu R, Jia P, Zhao Z (2020). 6mA-Finder: a novel online tool for predicting DNA N6-methyladenine sites in genomes. Bioinformatics.

[24] Xue T, Zhang S, Qiao H (2021). i6mA-VC: a multi-classifier voting method for the computational identification of DNA N6-methyladenine sites. Interdisciplinary Sciences: Computational Life Sciences.

[25] Khanal J, Lim DY, Tayara H, Chong KT (2021). i6mA-stack: a stacking ensemble-based computational prediction of DNA N6-methyladenine (6mA) sites in the Rosaceae genome. Genomics.

[26] Hasan MM, Basith S, Khatun MS, Lee G, Manavalan B, Kurata H (2021). Meta-i6mA: an interspecies predictor for identifying DNA N 6-methyladenine sites of plant genomes by exploiting informative features in an integrative machine-learning framework. Brief Bioinform.

[27] He S, Kong L, Chen J (2021). iDNA6mA-Rice-DL: a local web server for identifying DNA N6-methyladenine sites in rice genome by deep learning method. J Bioinform Comput Biol.

[28] Huang Q, Zhou W, Guo F, Xu L, Zhang L (2021). 6mA-Pred: identifying DNA N6-methyladenine sites based on deep learning. PeerJ.

[29] Le NQK, Ho Q-T (2022). Deep transformers and convolutional neural network in identifying DNA N6-methyladenine sites in cross-species genomes. Methods.

[30] Yang X, Ye X, Li X, Wei L (2021). iDNA-MT: identification DNA modification sites in multiple species by using Multi-task Learning based a neural Network Tool. Front Genet.

[31] Yu Y, He W, Jin J, Xiao G, Cui L, Zeng R, Wei L (2021). iDNA-ABT: advanced deep learning model for detecting DNA methylation with adaptive features and transductive information maximization. Bioinformatics.

[32] Hochreiter S, Schmidhuber J (1997). Long short-term memory. Neural Comput.

[33] LeCun Y, Boser B, Denker J, Henderson D, Howard R, Hubbard W, Jackel L. Handwritten digit recognition with a back-propagation network. Adv Neural Inf Process Syst. 1989; 2.

[34] He K, Zhang X, Ren S, Sun J. Deep Residual Learning for Image Recognition. In: 2016 IEEE Conference on Computer Vision and Pattern Recognition (CVPR): 27–30 June 2016 2016. Las Vegas. 2016. p. 770–778. 10.1109/CVPR.2016.90.

[35] Vaswani A, Shazeer N, Parmar N, Uszkoreit J, Jones L, Gomez AN, Kaiser L. Polosukhin I: attention is all you need. Adv Neural Inf Process Syst. 2017;30:5998–6008.

[36] Chen Z, Chen Y-Z, Wang X-F, Wang C, Yan R-X, Zhang Z (2011). Prediction of ubiquitination sites by using the composition of k-spaced amino acid pairs. PLoS ONE.

[37] Chen Z, Zhou Y, Song J, Zhang Z (2013). hCKSAAP_UbSite: improved prediction of human ubiquitination sites by exploiting amino acid pattern and properties. Biochim et Biophys Acta (BBA)-Proteins Proteom.

[38] Chen W, Tran H, Liang Z, Lin H, Zhang L (2015). Identification and analysis of the N6-methyladenosine in the Saccharomyces cerevisiae transcriptome. Sci Rep.

[39] Chen Z, Zhao P, Li F, Marquez-Lago TT, Leier A, Revote J, Zhu Y, Powell DR, Akutsu T, Webb GI (2020). iLearn: an integrated platform and meta-learner for feature engineering, machine-learning analysis and modeling of DNA, RNA and protein sequence data. Brief Bioinform.

[40] Jia C-Z, Zhang J-J, Gu W-Z (2016). RNA-MethylPred: a high-accuracy predictor to identify N6-methyladenosine in RNA. Anal Biochem.

[41] Qiu W-R, Xiao X, Chou K-C (2014). iRSpot-TNCPseAAC: identify recombination spots with trinucleotide composition and pseudo amino acid components. Int J Mol Sci.

[42] Chen W, Feng P-M, Lin H, Chou K-C (2013). iRSpot-PseDNC: identify recombination spots with pseudo dinucleotide composition. Nucleic Acids Res.

[43] Huang Y, He N, Chen Y, Chen Z, Li L (2018). BERMP: a cross-species classifier for predicting m6A sites by integrating a deep learning algorithm and a random forest approach. Int J Biol Sci.

[44] Chen Z, Zhao P, Li C, Li F, Xiang D, Chen Y-Z, Akutsu T, Daly RJ, Webb GI, Zhao Q (2021). iLearnPlus: a comprehensive and automated machine-learning platform for nucleic acid and protein sequence analysis, prediction and visualization. Nucleic Acids Res.

[45] Teng Z, Zhao Z, Li Y, Tian Z, Guo M, Lu Q, Wang G (2022). i6mA-Vote: cross-species identification of DNA N6-Methyladenine sites in Plant genomes based on Ensemble Learning with Voting. Front Plant Sci.

[46] Yu X, Hu J, Zhang Y. SNN6mA: improved DNA N6-methyladenine site prediction using siamese network-based feature embedding. Comput Biol Med. 2023; 107533. 10.1016/j.compbiomed.2023.107533. 10.1016/j.compbiomed.2023.107533 37793205

[47] Zhang Y, Liu Y, Xu J, Wang X, Peng X, Song J, Yu D-J (2021). Leveraging the attention mechanism to improve the identification of DNA N6-methyladenine sites. Brief Bioinform.

[48] Tahir M, Tayara H, Chong KT (2019). iDNA6mA (5-step rule): identification of DNA N6-methyladenine sites in the rice genome by intelligent computational model via Chou’s 5-step rule. Chemometr Intell Lab Syst.

[49] Park S, Wahab A, Nazari I, Ryu JH, Chong KT (2020). i6mA-DNC: prediction of DNA N6-Methyladenosine sites in rice genome based on dinucleotide representation using deep learning. Chemometr Intell Lab Syst.

[50] Lundberg SM, Lee S-I (2017). A unified approach to interpreting model predictions. Adv Neural Inf Process Syst.

[51] Nair AS, Sreenadhan SP (2006). A coding measure scheme employing electron-ion interaction pseudopotential (EIIP). Bioinformation.

[52] Jia C, Yang Q, Zou Q (2018). NucPosPred: Predicting species-specific genomic nucleosome positioning via four different modes of general PseKNC. J Theor Biol.

[53] Wu H, Zhang P, Ai Z, Wei L, Zhang H, Yang F, Cui L (2022). StackTADB: a stacking-based ensemble learning model for predicting the boundaries of topologically associating domains (TADs) accurately in fruit flies. Brief Bioinform.

